# Intergenogroup Recombination in Sapoviruses

**DOI:** 10.3201/eid1112.050722

**Published:** 2005-12

**Authors:** Grant S. Hansman, Naokazu Takeda, Tomoichiro Oka, Mitsukai Oseto, Kjell-Olof Hedlund, Kazuhiko Katayama

**Affiliations:** * National Institute of Infectious Diseases, Tokyo, Japan; † National Institute of Environmental Studies, Ehime, Japan; ‡ Swedish Institute for Infectious Disease Control, Solna, Sweden

**Keywords:** Sapovirus, intergenogroup, intragenogroup, recombination, gastroenteritis, research

## Abstract

Sapovirus, a member of the family *Caliciviridae*, is an etiologic agent of gastroenteritis in humans and pigs. Analyses of the complete genome sequences led us to identify the first sapovirus intergenogroup recombinant strain. Phylogenetic analysis of the nonstructural region (i.e., genome start to capsid start) grouped this strain into genogroup II, whereas the structural region (i.e., capsid start to genome end) grouped this strain into genogroup IV. We found that a recombination event occurred at the polymerase and capsid junction. This is the first report of intergenogroup recombination for any calicivirus and highlights a possible route of zoonoses because sapovirus strains that infect pig species belong to genogroup III.

## Abstract 2

This first report of intergenogroup recombination for any calicivirus highlights a possible route of zoonoses.

The family *Caliciviridae* contains 4 genera, *Sapovirus*, *Norovirus*, *Lagovirus*, and *Vesivirus*. The sapovirus (SaV) and norovirus (NoV) strains are etiologic agents of gastroenteritis in humans, although animals such as pigs, cows, and mice can also be infected. SaV strains were originally detected by using electron microscopy, but today the most widely used method is reverse transcription–polymerase chain reaction (RT-PCR), which has a high sensitivity (*1*). Based on the capsid gene sequence, SaV can be grouped into 5 distinct genogroups (GI to GV) (*2*). Human SaV belong to GI, GII, GIV, and GV, whereas pig SaV belongs to GIII. The SaV GI, GIV, and GV genomes are believed to each contain 3 main open reading frames (ORFs), whereas the SaV GII and GIII genomes each have only 2 main ORFs (*2*). ORF1 encodes nonstructural proteins and the capsid protein, while ORF2 and ORF3 encode proteins of yet-unknown functions. Using complete genome sequence analysis, we recently identified the first recombinant (intragenogroup) SaV strains (*3*). Two SaV strains, Mc10 and C12, both belonging to GII, were identified as recombinants. Phylogenetic analysis of the nonstructural region (i.e., genome start to capsid start) grouped Mc10 and C12 together in 1 GII cluster (or genotype), while the structural region (i.e., capsid start to genome end) grouped Mc10 and C12 into distinct GII genotypes. Evidence suggested that the recombination site occurred at the polymerase and capsid junction on ORF1. This site is highly conserved among SaV strains, which suggests that the recombination event occurs when nucleic acids of parental strains come into physical contact in infected cells, e.g., during copy choice recombination (*4*), as we have recently described with recombinant NoV strains (*5*).

## Materials and Methods

We compared the complete genome sequences of 11 SaV strains to analyze suspected novel recombinant SaV strains. For this study, we sequenced the complete genomes of 4 SaV strains (Mc2, SK15, Ehime1107, and SW278). The Mc2 strain was isolated from a child with gastroenteritis in Chiang Mai, Thailand, in 2000 (*6*); SK15 was isolated from an adult with gastroenteritis in Sakai, Japan, in 2001 (unpub. data); Ehime1107 was isolated from an adult with gastroenteritis in Matsuyama, Japan, in 2002 (unpub. data); and SW278 was isolated from an adult with gastroenteritis in Solna, Sweden, in 2003 (*7*). The complete genome sequences were amplified and sequenced as described earlier (*3*). Phylogenetic analysis was performed by using the Genetyx program (Genetyx for the Macintosh version 13.0.5, Genetyx Corp., Tokyo, Japan) and ClustalX (Version 1.82; available from http://www.embl.de/~chenna/clustal/darwin/). Trees were drawn by using njplot (for the Macintosh; available from http://pbil.univ-lyon1.fr/software/njplot.html).

## Results

**Figure 1:**
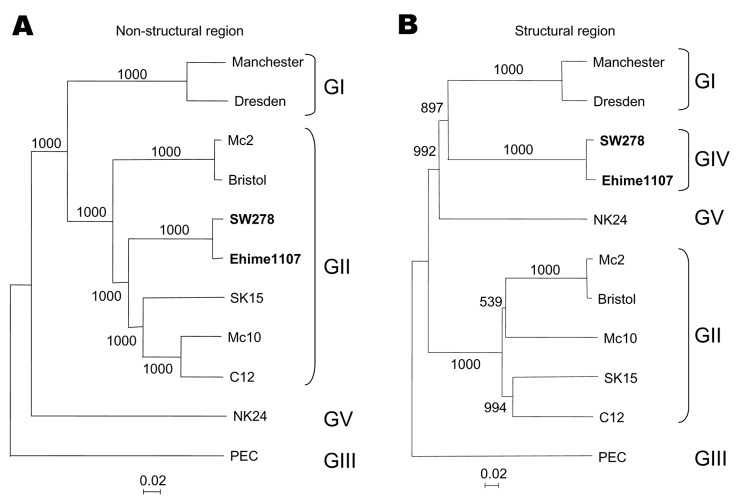
Phylogenetic analysis of (A) the nonstructural region (i.e., genome start to capsid start) and (B) the structural region (i.e., capsid start to genome end), showing the different genogroups. The numbers on each branch indicate the bootstrap values for the genotype. Bootstrap values >950 were considered significant for the grouping (10). The scale represents nucleotide substitutions per site. GenBank accession numbers are as follows: Mc10, AY237420; Manchester, X86560; Dresden, AY694184; SW278, DQ125333; Ehime1107, DQ058829; NK24, AY646856; C12, AY603425; Bristol, AJ249939; Mc2, AY237419; PEC, AF182760; and SK15, AY646855.

Based on the classification scheme of either the partial or complete capsid sequences in our previous studies, we grouped Manchester into GI; Bristol, Mc2, Mc10, C12, and SK15 into GII; PEC into GIII; and NK24 into GV (*6**,**8**,**9*). For this study and on the basis of the structural region (i.e., capsid start to genome end), we grouped Manchester into GI; Mc2, Bristol, Mc10, C12; and SK15 into GII; PEC into GIII; SW278 and Ehime1107 into GIV, and NK24 into GV (Figure 1). These genogroups were not maintained when we analyzed the nonstructural region (i.e., genome start to capsid start). We found that SW278 and Ehime1107 clustered into GII for the nonstructural region–based grouping but clustered into GIV for the structural region–based grouping. All genogroups were supported by bootstrap values (*10*), except for the structural region–based grouping of GI, which had a slightly lower value of 897. Nevertheless, these results indicate that the nonstructural region of SW278 and Ehime1107, i.e., a GII sequence, did not belong to a distinct genogroup, unlike their structural region, which belonged to a distinct genogroup (proposed as GIV). Comparisons of the complete genome sequences showed that SW278 and Ehime1107 shared >97% nucleotide identity and likely represented the same strain, although it was isolated from different countries; however, the lengths were different. Either SW278 or Ehime1107 had a 10-nucleotide insertion or deletion in the nontranslated region at the 3´ terminus. A number of closely matching partial sequences to SW278 and Ehime1107, which included both the polymerase and capsid gene, were available on the database, which indicates the circulation of similar strains in other countries.

**Figure 2:**
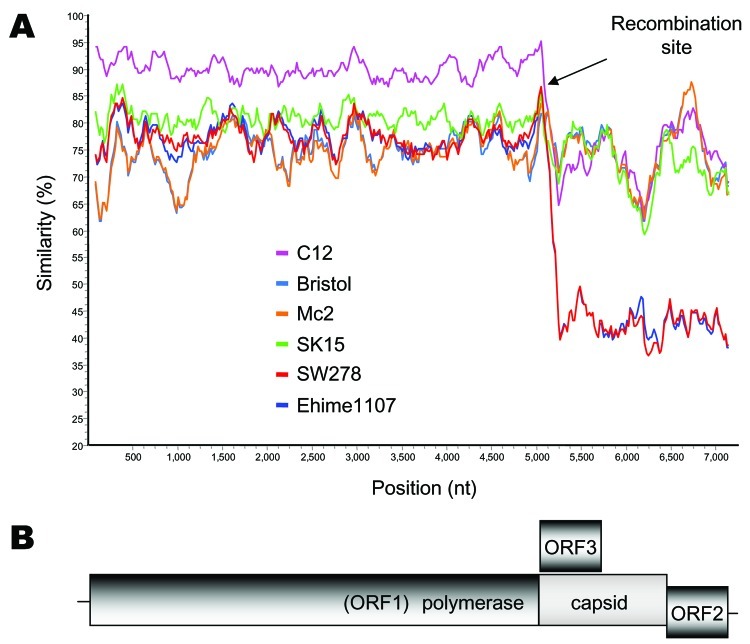
A) SimPlot analysis of 7 sapovirus (SaV) complete genome sequences. The Mc10 genome sequence was compared to C12, Bristol, Mc2, SK15, SW278, and Ehime1107 by using a window size of 100 bp with an increment of 20 bp. All gaps were removed. The recombination site is suspected to be located between the polymerase and capsid gene, as shown by the arrows. B) Genomic organization of the SaV SW278 and Ehime1107 strains.

We next used SimPlot (available from http://sray.med.som.jhmi.edu/SCRoftware/simplot/) with a window size of 100 and an increment of 20 bp (*11*) to further analyze these novel recombinant SW278 and Ehime1107 strains. We analyzed 7 complete genome SaV sequences. The Mc10 genome sequence was compared to C12, Bristol, Mc2, SK15, SW278, and Ehime1107. We observed a sudden drop in nucleotide similarity after the polymerase region for SW278 and Ehime1107 (Figure 2A). Nucleotide sequence analysis of the nonstructural region showed that SW278 and Ehime1107 shared between 74.0% to 77.6% nucleotide identity to the Mc2, C12, Mc10, and SK15 sequences, whereas analysis of the structural region showed that SW278 and Ehime1107 had only 54.0%–55.2% nucleotide identity to the Mc2, C12, Mc10, and SK15 sequences (Table); i.e., the nonstructural and structural regions of SW278 and Ehime1107 were ≈20% different. A similar result was observed with the nonstructural and structural regions of the already-established recombinant Mc10 and C12 strains, which had an 18.6% difference (*3*). When we analyzed the nonstructural and structural regions of Mc2 and SK15, we found only a 1.5% difference. Likewise, all other SaV strains generally maintained their nucleotide identities over the complete genome (Table). This result can be best explained as a recombination event at the polymerase and capsid junction for the SW278 and Ehime1107 strains, i.e., the nonstructural region originated from a GII strain, and the structural region originated from a strain belonging to another genogroup. The SaV GI, GIV, and GV genomes are predicted to encode an ORF3, whereas the SaV GII and GIII genomes have 2 main ORFs. We found that SW278 and Ehime1107 each had an ORF3, which is predicted to encode a yet-unknown protein of 161 amino acids. Notably, the structural region–based grouping showed that GI, GIV, and GV grouped in 1 major branch, while GII and GIII represented 2 other branches. These data provide further evidence of the intergenogroup recombination for SW278 and Ehime1107 strains.

**Table:** Nucleotide identity (%) among sapovirus strains*

Nonstructural region	Structural region									
	Ehime1107	SW278	Mc2	Mc10	C12	SK15	Dresden	Manchester	NK24	PEC
Ehime1107		96.9	54.6	55.1	54.4	55.2	58.3	58.8	58.3	51.0
SW278	97.5		54.6	55.2	54.0	54.7	58.1	58.3	58.3	50.8
Mc2	73.8	74.0		72.7	73.0	71.8	54.5	54.0	53.7	51.2
Mc10	77.5	77.6	74.4		71.5	71.1	55.3	54.6	55.2	50.7
C12	77.3	77.3	74.4	90.1		75.0	55.4	55.7	55.5	51.8
SK15	77.5	77.5	73.3	81.0	80.3		56.2	56.1	55.5	50.4
Dresden	62.6	62.7	63.3	63.0	63.1	62.4		92.9	57.3	52.5
Manchester	63.5	63.3	63.2	63.7	64.0	62.8	90.5		57.4	52.1
NK24	55.4	55.6	55.8	55.7	55.0	55.2	56.3	56.8		53.3
PEC	52.5	52.5	53.0	52.3	52.4	52.3	51.7	51.5	52.1	

*Values on the lower left represent the nonstructural region, i.e., genome start to capsid start; values on the upper right represent the structural region, i.e., capsid start to genome end.

**Figure 3:**
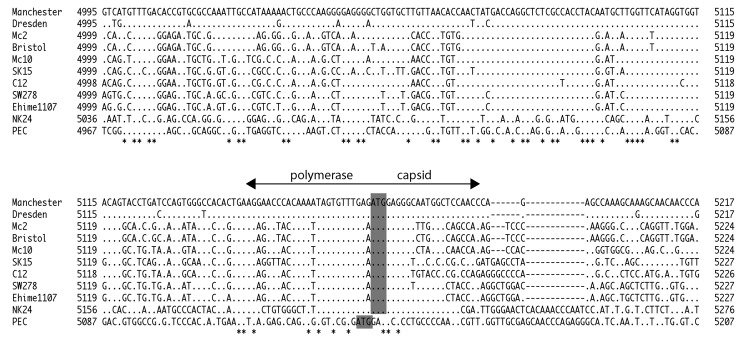
Nucleotide alignment of Manchester, Dresden, Mc2, Bristol, Mc10, SK15, C12, SW278, Ehime1107, NK24, and PEC sequences, showing the conserved polymerase and capsid junction. The asterisks represent conserved nucleotides. The shaded nucleotides represent the putative capsid start codons.

The SaV subgenomic RNA has not yet been identified, but for other caliciviruses the subgenomic RNA was identified (*12**–**14*). We recently provided evidence that the SaV viral protease was responsible for the cleavage of nonstructural and capsid proteins on ORF1 (*15*). Therefore, SaV replication may occur through at least 2 pathways: 1) the capsid protein was transcribed as a polyprotein on ORF1 and then cleaved, or 2) the capsid protein was transcribed as subgenomic RNA and then translated. The suspected recombination site occurred at the highly conserved polymerase and capsid junction for human SaV, as shown in Figure 3. Recombination is thought to occur when nucleic acids of the parental strains come into physical contact in infected cells, e.g., during copy choice recombination (*4*). These data suggest that recombinant SaV strains were formed either by full-length RNA template switching or full-length and subgenomic template switching.

## Discussion

These results are noteworthy because this is the first report of intergenogroup recombination for any calicivirus. These findings provide evidence that zoonoses could occur within the *Sapovirus* genus because strains that infect pig species belong to GIII. Furthermore, since the parent nonstructural region of SW278 and Ehime1107 has not yet been identified, we could not rule out that the parents of SW278 and Ehime1107 came from a strain that infects animals. We have conducted a number of molecular epidemiologic studies using broad-range primers and found that GIV strains were infrequently compared to other genogroups (*6**,**8**,**9**,**16**,**17*). This finding suggests 1) the emergence and/or recombination of GIV strains from an animal reservoir, 2) a lower prevalence of GIV strains, though a number of similar sequences were identified in the United States, or 3) our primers were less sensitive in detecting variant GIV sequences. Nevertheless, further complete genome analysis of other SaV strains is needed to identify other recombinant strains and determine the extent of recombination in the *Sapovirus* genus. Although we cannot easily pinpoint where and when the recombination event took place, screening of animals with primers designed against human SaV strains may also help identity the potential parental strain(s) of these 2 novel recombinants.

## Conclusions

To date, we have identified 4 different recombinant SaV strains, Mc10, C12, SW278, and Ehime1107. Collectively, these strains have 2 kinds of nonstructural sequences but 3 kinds of structural sequences (Figure 1). In addition, all nonstructural sequences belonged to GII. These data suggest that SaV could evade host immunity by readily changing their structural region (immunoreactive, i.e., capsid protein) and that GII strains (nonstructural–based grouping) are more capable of recombination than other genogroups. In 1999, Jiang et al. (*18*) identified the first naturally occurring human recombinant NoV, and several other strains were later described as recombinants (*5**,**6**,**19**–**21*). The site of genetic recombination for NoV was also between the polymerase and capsid genes. Human SaV and NoV strains cannot be cultivated, but the expression of the recombinant capsid protein (rVP1) in a baculovirus expression system results in the self-assembly of viruslike particles (VLPs) that are morphologically similar to native SaV. In a recent study, we genetically and antigenically analyzed 2 recombinant NoV strains (strains 026 and 9912-02F) (*17*). When polymerase-based grouping was performed, these 2 strains clustered together, but when capsid-based grouping was performed, these 2 strains belonged in 2 distinct genotypes. When we compared the cross-reactivity of these VLPs with an antibody enzyme-linked immunosorbent assay (ELISA), the titers of 026 antiserum against 026 and 9912-02F VLPs were 1:2,058,000 and 1:512,000, respectively, a 4-fold difference, whereas the titers of 9912-02F antiserum against 9912-02F and 026 VLPs were 1:1,024,000 and 1:128,000, respectively, an 8-fold difference. These results demonstrated that 026 and 9912-02F likely represented distinct antigenic types, which correlated with the genetic analysis. The expression of SaV VLPs is also needed to determine the cross-reactivity among these recombinant strains, although our results have shown that GI and GV VLPs (capsid-based grouping) were antigenically distinct by an antibody and antigen ELISA (*22*), which suggests that these 2 recombinant strains are also antigenically distinct from GII strains. And finally, these results will have a major influence on the future phylogenetic classification of SaV strains. Therefore, the genetic classification of SaV strains needs to be addressed, and a consensus of prototype strains representing genogroups and genotypes should be established to avoid further grouping conflicts.
